# Association of vitamin D status with socio-demographic factors in Calgary, Alberta: an ecological study using Census Canada data

**DOI:** 10.1186/1471-2458-13-316

**Published:** 2013-04-08

**Authors:** Christopher Naugler, Jianguo Zhang, Dan Henne, Paul Woods, Brenda R Hemmelgarn

**Affiliations:** 1 Department of Pathology and Laboratory Medicine, University of Calgary, Calgary, Alberta, Canada; 2 Department of Family Medicine, University of Calgary, Calgary, Alberta, Canada; 3 Calgary Laboratory Services, Calgary, Alberta, Canada; 4 C414, Diagnostic and Scientific Centre, 9, 3535 Research Road NW, Calgary, AB T2L 2K8, Canada; 5 Department of Medicine, University of Calgary, Calgary, Alberta, Canada

**Keywords:** Vitamin D, Ecological study, Sociodemographic factors

## Abstract

**Background:**

Low 25-hydroxyvitamin D levels are a global health problem with northern countries such as Canada at particular risk. A number of sociodemographic factors have been reported to be associated with low vitamin D levels but prior studies have been limited by the ability of the researchers to gather this data directly from clinical trial participants. The purpose of this study was to use a novel methodology of inferring sociodemographic variables to evaluate the correlates of vitamin D levels in individuals dwelling in the City of Calgary, Alberta, Canada.

**Methods:**

We utilized data on vitamin D test results from Calgary Laboratory Services between January 1 2010 and August 31 2011. In addition to vitamin D level, we recorded age, sex, and vitamin D testing month as individual-level variables. We inferred sociodemographic variables by associating results with census dissemination areas and using Census Canada data to determine immigration status, education, median household income and first nations status as clustered variables. Associations between vitamin D status and the individual- and dissemination area-specific variables were examined using the population-averaged regression model by a generalized estimating equations approach to account for the clustering in the data.

**Results:**

158,327 individuals were included. Age, sex, month of vitamin D testing (at an individual level), and education, immigrant status, first nations status and income (at an aggregate level) were all statistically significant predictors of vitamin D status.

**Conclusions:**

Vitamin D status was associated with a number of sociodemographic variables. Knowledge of these variables may improve targeted education and public health initiatives.

## Background

Vitamin D has received considerable interest from the medical community and the public at large because of its importance in the maintenance of health [1-6] combined with the finding of widespread global deficiency [7-12]. Among the major micronutrients, vitamin D is unique in that the primary source is solar ultraviolet B radiation [13] (which converts 7-dehydocholesterol to vitamin D₃ in the skin), with oral supplementation representing an important secondary source [14]. These characteristics make vitamin D deficiency an important target for public health interventions.

High latitude countries such as Canada may be especially vulnerable to vitamin D deficiency because of lower ultraviolet radiation levels [13-23]. Indeed findings of a Canadian cross sectional survey suggested that as many as three million Canadians have inadequate vitamin D levels and 1.1 million Canadians are vitamin D deficient (defined as a serum vitamin D level ≤27.5 nmol/L; to convert 25-hydroxyvitamin D levels from SI to conventional units divide by 2.496) [17]. Other specific sociodemographic factors have been reported to be associated with low vitamin D levels including advanced age and/or residence in a nursing home [24], cultural factors such as skin covering [25,26], aboriginal ancestry [27], low dietary intake of milk [17], darker skin pigmentation and/or non-white ethnicity [14,17,18,21,28], obesity [18,29], and lower education [30].

However there is scope for additional large scale examination of the sociodemographic correlates of vitamin D status, particularly with regards to aboriginal ancestry, education and the largely unstudied variable of household income, as these variables have received only limited attention in prior studies [28,30]. In this study we combined a secondary analysis of laboratory test results with aggregate census Canada data to determine sociodemographic factors independently associated with serum vitamin D levels in a large sample of individuals in Calgary, a northern Canadian city of Alberta province. Although prior researchers have utilized geospatial mapping to infer health-related variables [31-34], this approach is novel in that it is being applied for the first time to vitamin D data. We hypothesized that spatial variance in sociodemographic factors within the city of Calgary will be associated with spatial variance in mean 25-hydroxyvitamin D levels, and that these variances can be used to infer sociodemographic associations with 25-hydroxyvitamin D level.

The recent Institute of Medicine (IOM) report on Dietary Reference Intakes for vitamin D for Canada and the USA, suggests that for the skeletal benefits of vitamin D, a 25-hydroxyvitamin D level level of 50 nmol/L reflects adequate vitamin D intake for 97.5% of the population [35]. In our analysis, we therefore defined 25-hydroxyvitamin D ‘sufficiency’ as a serum level of greater then or equal to 50 nmol/L.

## Methods

### Ethics statement

The study protocol was approved by the University of Calgary Conjoint Health Review Ethics Board (Ethics ID 23919).

### Study population and data sources

We undertook this observational study combining laboratory data with clustered (census dissemination area level) variables obtained from the 2006 Canadian Census of Population. The study population consisted of adults 25 years of age and older who underwent vitamin D testing at Calgary Laboratory Services (CLS) between January 01, 2010 and August 31, 2011. CLS is the sole provider of laboratory testing to Calgary, Alberta and the surrounding areas (approximate population 1.4 million). All 25-hydroxyvitamin D tests were performed as part of routine patient care and were analyzed in a single laboratory using the LIASON 25-hydroxyvitamin D Total assay (Diasorin, Ltd.) on Roche modular analyzers. The lower limit of detection of this assay was 10 nmol/L. For the purpose of analysis, values <10 nmol/L were recorded as 10 nmol/L. Quality control is performed daily on these analyzers. Quality assurance is performed through subscription to the Vitamin D External Quality Assessment Scheme (DEQAS). For the year 2012 our lab's average bias from our method mean (DiaSorin Liaison Total) for all samples was less than 5%, with no individual result being more than 10% from the method mean. The inter-assay coefficient of variation was 4.2% at 42 nmol/l and 2.9% at 130 nmol/L. Intra-Assay coefficient of variations from our daily quality controls was 8.5% at 50 nmol/L, 8.3% at 85 nmol/L and 9.6% at 265 nmol/L.

To avoid pseudoreplication, each patient was included only once in the analysis. If more than one record existed for the same individual, one record was chosen at random. An alternative approach would have been to choose the first available test result for each individual; this may have excluded some measurements taken to determine response to supplementation. However, because of the limited time frame covered by our data and our inability to determine if patients were taking supplements, we opted to use one test result taken at random for each patient. For each vitamin D test record the following information was extracted from the laboratory information system: 25-hydroxyvitamin D level, age, sex, month of testing, and health care number. Health care number was then used as a linking variable to subject postal codes from an Alberta Health Services database. The postal codes were converted to their corresponding census dissemination area and geographic coordinates using a Statistics Canada Postal Code Conversion File. Census Dissemination Areas (CDA) are the lowest geographic level in the Canadian Census and consist of geographical groupings of 300–700 people. We considered only individuals residing within the City of Calgary and so census dissemination areas outside of the city limits were removed from the dataset. Finally, 25-hydroxyvitamin D levels greater than 300 nmol/L were deemed as extreme values and removed.

The resulting dataset was plotted onto a map of the city of Calgary using ArcGIS v. 9.3 software at the Spatial and Numeric Services Department of the University of Calgary Library. Following mapping of the individual records, sociodemographic variables for each dissemination area within the city of Calgary were imported from Statistics Canada data obtained from the 2006 Canadian Census [36]. These data were then joined as clustered variables at the level of census dissemination area from the 2006 Canada Census. The following dissemination area variables were added to the dataset: percent of individuals of aboriginal descent, median household income, percent of individuals born outside of Canada, and percent of individuals over age 25 with at least some post-secondary education. This combined data set was then used to examine associations between 25-hydroxyvitamin D level and the various sociodemographic variables.

### Statistical analysis

Data were presented as mean and standard deviation (SD), or median and interquartile range (IQR), when appropriate, for continuous variables; and, as frequencies and proportions for categorical variables. Given the correlated (clustered) nature of the data (i.e. individuals were clustered within census dissemination areas where they lived in as the data was collected), we used the SAS macro % RCS_Reg as detailed in [37] to fit the marginal (population-averaged) generalized estimating equations (GEE) regression model to account for the clustering in the data, while using restricted cubic spline (RCS) functions (i) to visually and statistically check the assumption of linearity of the association between each of the continuous predictors and the outcome, and, (ii) to graphically characterize and quantify the association when the latter assumption is not valid [37]. The number of knots for the splines of each continuous predictor was chosen according to the information criteria (QIC and QICu, the smaller the better). The final GEE model included each of the continuous predictors with their restricted cubic splines, as well as gender and month of vitamin D testing as categorical variables. All reported P values were two-sided, and considered as significant if < 0.05.

## Results and discussion

**Figure 1:**
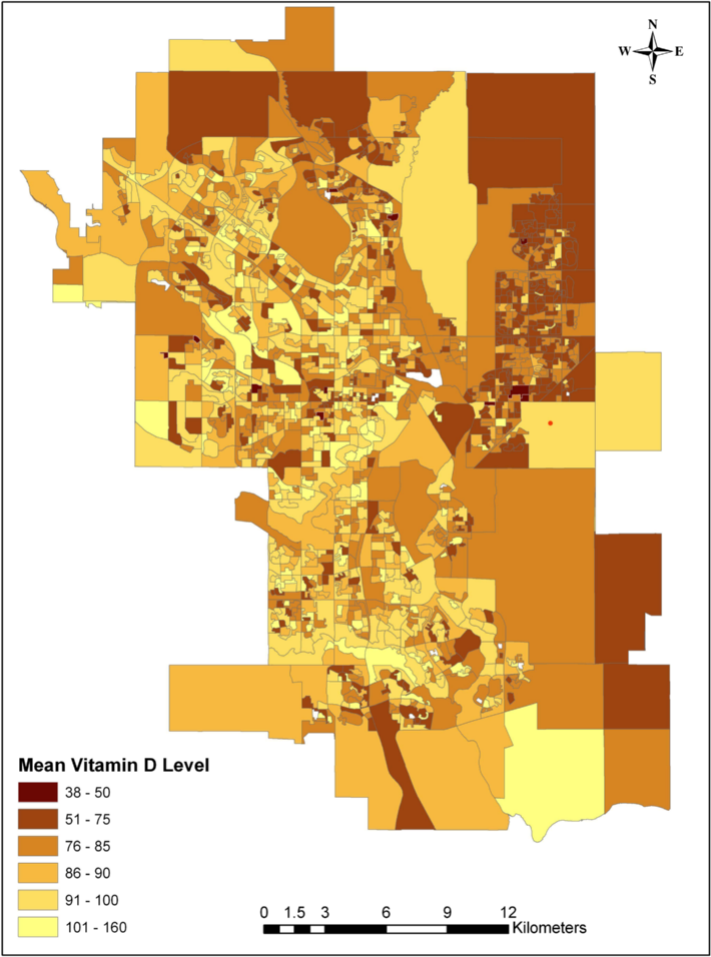
**Mean vitamin D levels in Calgary Alberta.** Map of the city of Calgary showing mean vitamin D levels (nmol/L) by census dissemination area (1986 areas with a total of 158,327 individuals).

**Figure 2:**
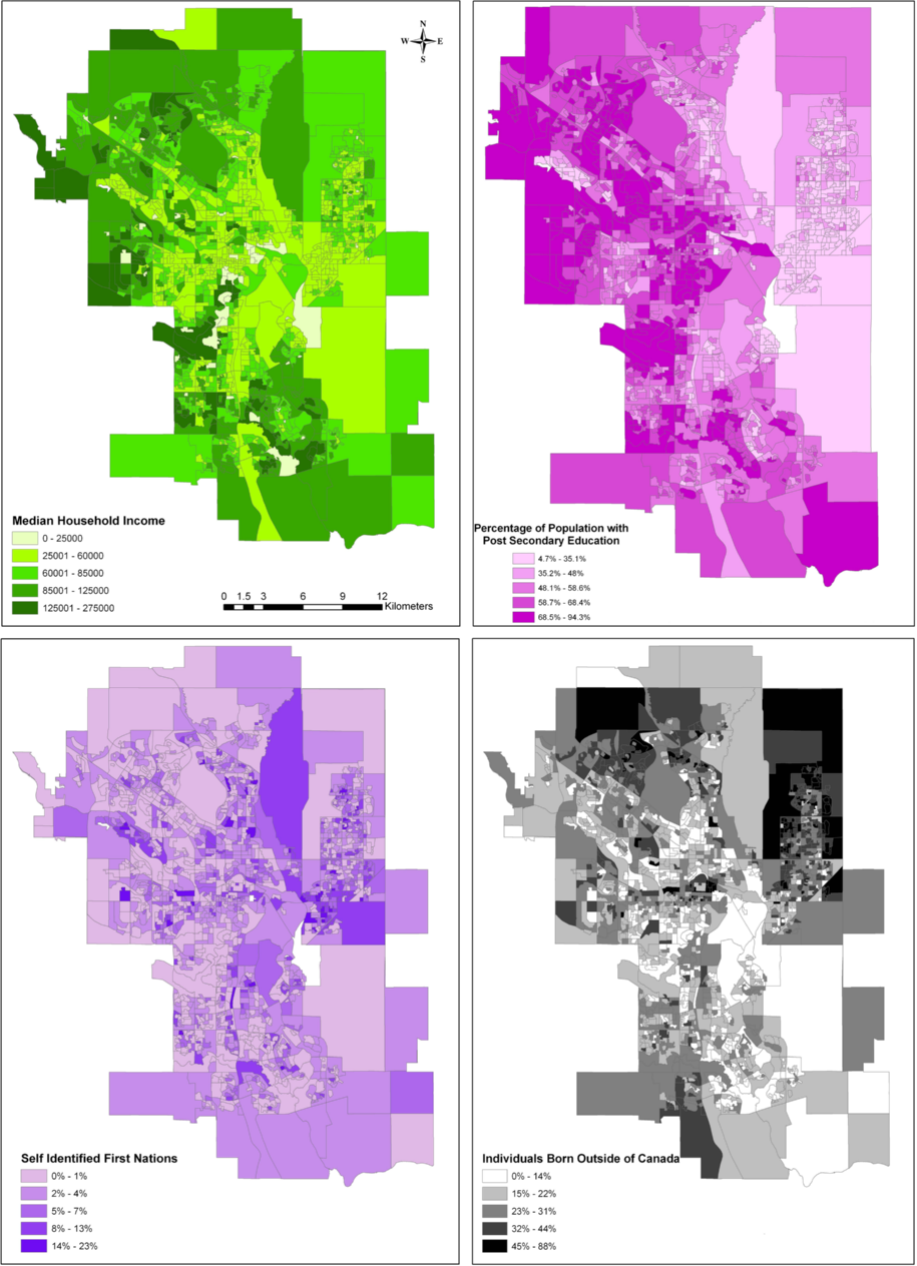
**Baseline sociodemographic variables for the city of Calgary.** Variables given by census dissemination area (data from the 2006 Canada Census).

Data were abstracted from a total of 1986 census dissemination areas within the City of Calgary, with a median number of 111 (IQR: 68–243) individuals being surveyed in each area. Outlier 25-hydroxyvitamin D levels greater than 300 were excluded from the analysis (119 cases) resulting in individual level data for a total of 158,327 patients. The majority (64.9%) were female, and most (73.9%) had a vitamin D level greater than or equal to 50 nmol/L. Median 25-hydroxyvitamin D levels as well as the proportion of individuals falling below the Institute of Medicine categories of vitamin adequacy [35] are given in Table 1. Associations of 25-hydroxyvitamin D sufficiency and the individual- and census dissemination area-level variables are summarized in Table 2. The mean vitamin D level per dissemination area is shown in Figure 1. As this map shows, higher average levels of vitamin D tended to be found in the inner city neighborhoods while the lowest levels tended to cluster in the far north and northeast. Chloropleths of median household income, percent of the population with at least some post-secondary education, percent of individuals of aboriginal decent and percent of individuals born outside of Canada (Figure 2) revealed some broad sociodemographic trends within the city. Notably, higher incomes and higher education levels tended to cluster in inner city neighborhoods and in the northwest (near the University of Calgary), while immigrants tended to be found in the north and northeast. The distribution of aboriginal people tended to be highest in the east and in certain inner city neighborhoods.

**Table 1:** **Median 25 hydroxyvitamin D level for the Calgary population studied, and the proportion of the population below levels representing the Institute Of Medicine’s**[31]**categories of vitamin adequacy: deficiency (<30 nmol/L), estimated average requirement (40 nmol/L) and recommended dietary allowance (50 nmol/L)**

	**Males**	**Females**
**Median 25 hydroxyvitamin D level**	65	71
**Proportion of individuals <30 nmol/L**	0.10	0.07
**Proportion of individuals <40 nmol/L**	0.19	0.15
**Proportion of individuals <50 nmol/L**	0.30	0.24

**Table 2:** Data summary of the individual- and census dissemination area-level variables included in the analysis, stratified by the presence of vitamin D insufficiency

**Variable**	**Vitamin D insufficient (< 50 nmol/L)**	**Vitamin D sufficient (≥ 50 nmol/L)**	**Total**
	**(N=41,401)**	**(N=116,926)**	**(N=158,327)**
**Age (years)**			
Mean (SD)	47.7 (14.4)	53.6 (15.4)	52.1 (15.4)
**Gender**, n (%)			
Female	24262 (58.6)	78417 (67.1)	102679 (64.9)
**Month of Vitamin D testing**, n (%)			
January	4084 (9.9)	10615 (9.1)	14699 (9.3)
Feburary	4210 (10.2)	10996 (9.4)	15206 (9.6)
March	6025 (14.6)	12657 (10.8)	18682 (11.8)
April	4371 (10.6)	11153 (9.5)	15524 (9.8)
May	3999 (9.7)		12245 (10.5)	16244 (10.3)
June	4345 (10.5)	12409 (10.6)	16754 (10.6)	
July	2939 (7.1)	10736 (9.2)	13675 (8.6)	
August	2863 (6.9)	10912 (9.3)	13775 (8.7)	
September	2015 (4.9)	6723 (5.7)	8738 (5.5)	
October	2265 (5.5)	6745 (5.8)	9010 (5.7)	
November	2277 (5.5)	6618 (5.7)	8895 (5.6)	
December	2008 (4.9)	5117 (4.4)	7125 (4.5)	
**Median household income of the dissemination area ($CDN)**				
Median (IQR^1^)	70690 (52623–87577)	73831 (55649–96393)	73264 (54982–94482)	
**Percent of individuals in the dissemination area not born in Canada**				
Median (IQR)	25.6 (17.3-37.1)	21.0 (14.1-29.9)	22.3 (14.8-31.9)	
**Percent of aboriginals in the dissemination area**				
Median (IQR)	1.7 (0.0 - 3.4)	1.6 (0.0 - 3.0)	1.6 (0.0 - 3.1)	
**Percent of individuals >= 25 years old in the dissemination area with an university degree**				
Median (IQR)	49.0 (35.9-62.6)	56.9 (43.9-67.9)	55.7 (41.1 - 66.7)	

^1^ IQR=interquartile range.

**Figure 3:**
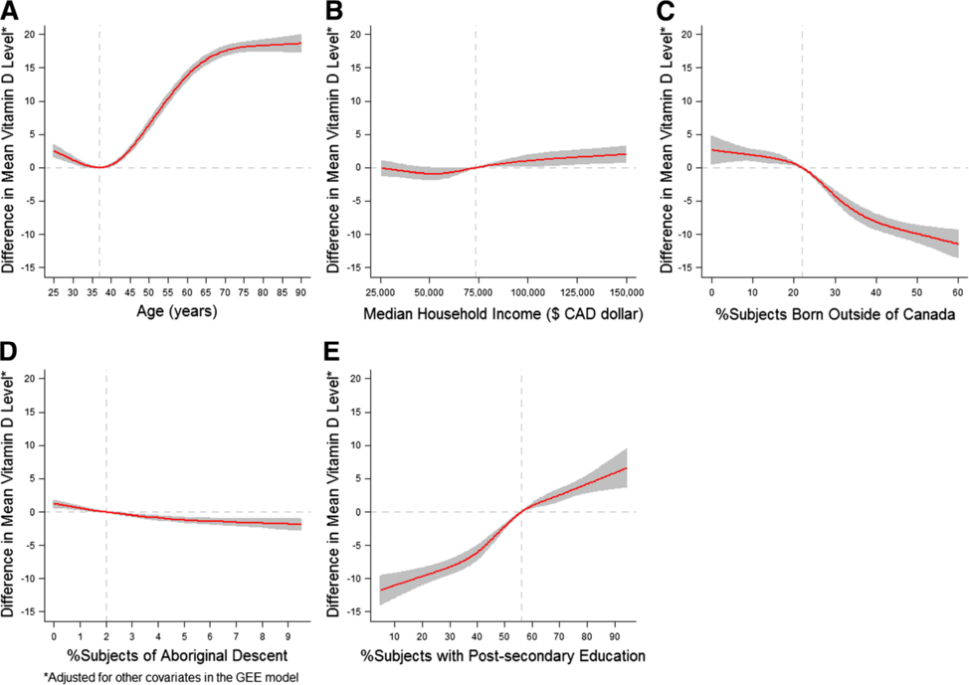
**Adjusted associations of vitamin D level (nmol/L).** (**A**) age (reference value = 37 years); (**B**) median household income (reference value = $CDN 73,264, the median); (**C**) percentage of individuals who were not born in Canada (reference value = 22%, the median); (**D**) percentage of Aboriginal people (reference value = 2%, the median); (**E**) percentage of individuals ≥25 years old who had at least some post-secondary education (reference value = 56%, the median). Age, median household income, percentage of immigrants, and percentage of subjects with some post-secondary education were coded using restricted cubic spline (RCS) functions with five knots located at the 5th, 25th, 50th, 75th, and 95th percentiles of their distribution, and percentage of aboriginal people was coded using the 5th, 50th and 95th percentiles of its distribution. Y-axis represents the difference in mean vitamin D level between individuals with any value of the continuous predictor (as indicated on the x-axis) and individuals with the reference value (as indicated by the vertical dashed line). Shaded area represents the 95% confidence band. Horizontal dashed line indicates the zero difference in mean 25 hydroxyvitamin D level.

Table 3 shows the results from the population-averaged GEE model investigating the association between 25-hydroxy vitamin D status and each of the predictors. The test for the overall association between 25-hydroxyvitamin D level and each of the continuous predictors was significant (all P-values < 0.0001). The test for non-linear association was also significant, which means that the association between 25-hydroxyvitamin D level and each of the continuous predictors was significantly not linear (p-value for linearity test was <0.0001, 0.0295, <0.0001, 0.009 and 0.0002 for age, median household income, percent of individuals born outside of Canada, percent of individuals of aboriginal decent and percent of population with at least some post-secondary education, respectively). Figure 3 graphically displays the nonlinear relationships between 25-hydroxyvitamin D level and each of the continuous predictors, with each graph showing the adjusted difference in mean 25-hydroxyvitamin D level (as indicated on the y-axis) between individuals with any value of the corresponding continuous predictor (as indicated on the x-axis) and individuals with the specified reference value (as described in Figure 3), when the other continuous variables plus gender and month of 25-hydroxyvitamin D testing were adjusted for. Interestingly, as shown in Figure 3, when other covariates were controlled in the generalized estimating equation model, median household income and percent of individuals of aboriginal descent showed only very modest associations with 25-hydroxyvitamin D level, although the associations were statistically significant. In contrast, education showed a clear relationship in that the mean levels of 25-hydroxyvitamin D were higher in census dissemination areas that had a greater proportion of individuals with at least some post-secondary education. Similarly, census dissemination area immigrant status was related to lower census dissemination area levels of 25-hydroxyvitamin D. The function for age was more complex with a nadir at age around 35 years then a steady increase in vitamin D level before leveling off around age 65 years (Figure 3).

**Table 3:** Estimates from the population-averaged GEE model investigating associations with vitamin D level

**Parameter**	**Estimate**	**Standard error**	**P-value**	**Overall p-value**
**Intercept**	64.5883	2.4201	<.0001	<.0001
**Age (years)**				<.0001
age_RCS_lin	−0.2738	0.0422	<.0001	
age_RCS_S1	0.0015	0.0001	<.0001	
age_RCS_S2	−0.0030	0.0004	<.0001	
age_RCS_S3	0.0008	0.0004	0.0823	
**Median household income ($CDN 10000)**				<.0001
income_RCS_lin	−0.4078	0.2350	0.0826	
income_RCS_S1	0.0543	0.0263	0.0392	
income_RCS_S2	−0.1270	0.0793	0.1092	
income_RCS_S3	0.0824	0.0855	0.3352	
**Percentage of immigrants**				<.0001
percentimmi__RCS_lin	−7.6190	9.4844	0.4218	
percentimmi__RCS_S1	−172.823	567.4589	0.7607	
percentimmi__RCS_S2	−1122.79	1547.330	0.4681	
percentimmi__RCS_S3	3152.879	1511.398	0.0370	
**Percentage of aboriginal people**				<.0001
percentabor__RCS_lin	−68.5534	15.3580	<.0001	
percentabor__RCS_S1	13868.36	5311.648	0.0090	
**Percentage of subjects with some post-secondary education**				<.0001
percentuniv_RCS_lin	13.6596	4.2690	0.0014	
percentuniv_RCS_S1	237.8126	69.0070	0.0006	
percentuniv_RCS_S2	−842.990	263.7487	0.0014	
percentuniv_RCS_S3	1090.294	509.4453	0.0323	
**Gender**				<.0001
Female	7.4674	0.1930	<.0001	
Male	0.0000	0.0000	.	
**Month of vitamin D testing**				<.0001
December	−1.3870	0.5024	0.0058	
November	0.1357	0.4583	0.7672	
October	−1.3138	0.4593	0.0042	
September	−0.1319	0.4590	0.7738	
August	4.7383	0.4252	<.0001	
July	3.7308	0.4142	<.0001	
June	0.5034	0.3959	0.2035	
May	3.5073	0.4330	<.0001	
April	0.6403	0.4129	0.1209	
March	−3.6969	0.4045	<.0001	
February	0.4483	0.4108	0.2751	
January	0.0000	0.0000	.	

Note: Age, median household income, percentage of immigrants, and percentage of subjects with some post-secondary education were coded using restricted cubic spline (RCS) functions with five knots located at the 5th, 25th, 50th, 75th, and 95th percentiles of their distribution, and percentage of aboriginal people was coded using the 5th, 50th and 95th percentiles of its distribution. The variables listed underneath each of the continuous variables are their corresponding spline variables (e.g., age_RCS_lin, age_RCS_S1, age_RCS_S2, and age_RCS_S3 are the 4 spline variables of age).

In addition, gender had also a statistically significant association with 25-hydroxyvitamin D level, with females exhibiting on average a 25-hydroxyvitamin D level 7.5 nmol/L (95% CI: 7.1 - 7.9 nmol/L, p < 0.0001) higher than males. We observed that mean vitamin D level differed according to month (Table 1), although the investigation of these differences was not one of purposes of this paper.

Overall, 26% of individuals in our study were 25-hydroxyvitamin D deficient or insufficient (serum 25-hydroxyvitamin D ≤ 50 nmol/L), compared with 20% of individuals in the Canadian multicentre osteoporosis study (CAMOS) [18]. The difference may be due to the differing ages of the subjects in these studies: whereas the (CAMOS) only considered adults over age 35, we included individuals age 25 and older. However, it should also be noted that the CAMOS subjects were recruited by random selection from the population living within 50 km of the centre of the Canadian cities studied, while the present study is not a random selection of the Calgary population. It is also possible that the difference may be due to different proportions of non-white subjects in each study. The 2007–2009 Canadian Health Measures Survey [17] reported a relationship between age and 25-hydroxyvitamin D level with the 20–39 age groups for both women and men showing the lowest vitamin D levels. This is also consistent with our findings.

Our observation of a nadir in 25- OH vitamin D levels in young adults may seem at odds with the widely held perception that vitamin D deficiency is principally a problem of older individuals [24,38-41]. It is possible that the low levels of 25-hydroxyvitamin D in the elderly may not be related to age per se but to other correlated variables such as education. Alternatively, it is possible that older individuals or those with greater post-secondary education are achieving higher 25-hydroxyvitamin D levels through supplementation. The inferred relationship we found between immigrant status and lower 25-hydroxyvitamin D levels is likely due to immigrant status being a proxy measure for increased skin pigmentation, a well-known risk factor for 25-hydroxyvitamin D deficiency [17,19,21,42-44]. The previously reported lower 25-hydroxyvitamin D level in Canadian aboriginals [27] is only weakly supported by our data. Rather, our findings suggest that the reported association may be due to other correlated factors such as age and education level. We confirmed this by comparing the crude association between the percentage of aboriginal people and vitamin D levels with the adjusted association between percentage of aboriginal people and vitamin D levels. The resulting crude association was stronger (i.e., showed a steeper slope between percentage of aboriginal people and vitamin D levels) than the adjusted association, suggesting that the association observed between percentage of aboriginal people and vitamin D could be indeed explained by age and education level (analysis not shown).

Interestingly, income demonstrated a very minor association with 25-hydroxyvitamin D level. This was somewhat unexpected as income has been shown to be an important health predictor in other contexts [45]. Finally, the association between 25-hydroxyvitamin D level and post-secondary education is consistent with a recent Finnish study [30]. It is tempting to speculate that this relationship may be related to increased use of vitamin D supplements among individuals with higher education [46].

Our study should be interpreted in light of its limitations. Firstly, because we relied on the secondary use of laboratory results combined with Census Canada data, we were unable to capture potentially important information such as dietary milk consumption [17,18,47], body mass index [18,29] and 25-hydroxyvitamin D supplementation [18]. Nor did we have access to a surrogate for 25-hydroxyvitamin D status such as parathyroid hormone level. A second potential limitation concerns the changing nature of the demographic make-up of the city of Calgary. Although we used 25-hydroxyvitamin D results from 2010 to 2011, the census Canada data we used was from the 2006 census. If there was a considerable demographic shift within a given census dissemination area in the intervening few years, this could have affected the clustered variables we used. Third, although dramatic differences in mean 25-hydroxyvitamin D levels existed among census dissemination areas, we cannot exclude the possibility of a confounding effect if the probability of individuals with low or high vitamin D being tested also varied with these socio-demographic variables. However, our large sample size should mitigate against this effect. Finally, this study shares the potential weakness of all studies utilizing ecological data in that the inferences regarding group level variables may not necessarily reflect individual level variables.

## Conclusions

In this study we examined the associations between a number of sociodemographic variables and 25-hydroxyvitamin D level using a combination of secondary clinical data with individual level variables and clustered variables derived from Census Canada data. We found that the mean levels of 25-hydroxyvitamin D for the city of Calgary varied widely by census dissemination area and that the predominant predictors of this variation seemed to be age, education level and immigration status among the variables considered in this study.

## Competing interests

The authors declare no relevant competing interests.

## Authors’ contributions

CN conceived of the study. All authors contributed to the study design. CN collected the data. CN and JZ performed he analyses. CN drafted the manuscript. All authors contributed to revisions, read and approved the final manuscript.

## Pre-publication history

The pre-publication history for this paper can be accessed here:

http://www.biomedcentral.com/1471-2458/13/316/prepub
